# Comparative performances of machine learning algorithms in radiomics and impacting factors

**DOI:** 10.1038/s41598-023-39738-7

**Published:** 2023-08-28

**Authors:** Antoine Decoux, Loic Duron, Paul Habert, Victoire Roblot, Emina Arsovic, Guillaume Chassagnon, Armelle Arnoux, Laure Fournier

**Affiliations:** 1 Université Paris Cité, PARCC UMRS 970, INSERM, Paris, France; 2 Unité de Recherche Clinique, Center d’Investigation Clinique 1418 Épidémiologie Clinique, Université Paris Cité, AP-HP, Hôpital Européen Georges Pompidou, INSERM, Paris, France; 3 Department of Radiology, Hôpital Fondation Ophtalmologique Adolphe de Rothschild, Paris, France; 4 Imaging Department, Hôpital Nord, APHM, Aix Marseille University, Marseille, France; 5 Aix Marseille Univ, LIIE, Marseille, France; 6 Department of Radiology, Université Paris Cité, AP-HP, Hôpital Cochin, Paris, France; 7 Department of Radiology, Université Paris Cité, AP-HP, Hôpital Européen Georges Pompidou, PARCC UMRS 970, INSERM, Paris, France

**Keywords:** Computational biology and bioinformatics, Image processing, Machine learning

## Abstract

There are no current recommendations on which machine learning (ML) algorithms should be used in radiomics. The objective was to compare performances of ML algorithms in radiomics when applied to different clinical questions to determine whether some strategies could give the best and most stable performances regardless of datasets. This study compares the performances of nine feature selection algorithms combined with fourteen binary classification algorithms on ten datasets. These datasets included radiomics features and clinical diagnosis for binary clinical classifications including COVID-19 pneumonia or sarcopenia on CT, head and neck, orbital or uterine lesions on MRI. For each dataset, a train-test split was created. Each of the 126 (9 × 14) combinations of feature selection algorithms and classification algorithms was trained and tuned using a ten-fold cross validation, then AUC was computed. This procedure was repeated three times per dataset. Best overall performances were obtained with JMI and JMIM as feature selection algorithms and random forest and linear regression models as classification algorithms. The choice of the classification algorithm was the factor explaining most of the performance variation (10% of total variance). The choice of the feature selection algorithm explained only 2% of variation, while the train-test split explained 9%.

## Aims and objectives

Radiomics can be defined as the quantitative extraction of a high number of features from medical images for discovery of new predictive, diagnostic or prognostic imaging biomarkers of disease. Radiomics enables the non-invasive extraction of information invisible to the human eye from medical images using machine learning techniques and has shown promising results. However, the lack of standards hinders the use of radiomics biomarkers in a clinical setting^1^.

A radiomics study is structured in five steps: cohort constitution and imaging acquisition, segmentation of the region of interest (ROI), feature extraction, modeling and external validation on an (ideally) independent dataset^2^.

The modeling phase itself relies on two distinct steps: feature selection and prediction. For each step, many different methods and algorithms are available, which leads to a large number of possible combinations. To date, no strategy or recommendation has emerged on which algorithm(s) should be used preferentially when performing radiomics. Some teams have therefore chosen to test simultaneously different algorithms when performing studies, as it is believed that the algorithms which provided the best results depend of the scenario^3^. However, testing a large number of strategies when performing radiomics on a given dataset increases the risk of false discoveries. Therefore, it may be desirable to use a smaller number of selected models to increase chances of meaningful results.

Even if there are some initiatives to issue recommendations such as the Radiomics Quality Score^2^ or the Checklist for Artificial Intelligence in Medical Imaging (CLAIM)^4^, these recommendations are not well followed. For example, out of the 69 machine learning studies on diagnosis or prognosis of Covid-19 investigated by Roberts et al^5^, only 25 got a RQS above 6 out of 36. These results are supported by Spadarella et al.’s review^6^, which obtained a median RQS of 21% (7,5) for 44 radiomics studies. This is a significant issue, as poor methodological choices at different steps of the studies could lead to biased results. Bias could be introduced as early as the cohort constitution step if the distribution of the training dataset is different of the target population^7^. It can also be introduced by operator variability during the annotation of the dataset. Joskowicz et al^8^ showed on 3193 CT segmentations that the mean volume overlap variability between two observers was 37%. This variability can prevent some radiomics features from being reproducible. Also, ML algorithms could overfit or provided ill-estimated performances. Varoquaux et al.’s^9^ experiments on neuroimaging datasets reveal that a study sample size of one hundred leads to ± 10% errors in prediction accuracy. Conversely, Roelofs et al.’s study^10^ on Kaggle competitions showed that overfitting can be prevented by large enough test samples. Roelofs considered 10,000 examples as the minimum to protect against overfitting. 

The purpose of this study was to focus on the modeling phase of the radiomics workflow to determine whether some – and which – combination of algorithms could give the best and most stable performances in radiomics studies, regardless of datasets. This would serve to guide users in their choice of modeling strategies when performing radiomics. A secondary objective was to determine the main factors impacting the models’ performances.

## Materials and methods

### Materials

In order to estimate the impact of the choice of the methods and algorithms on models’ performances, we used ten datasets from various radiomics studies previously published or submitted^11–14^. This study adhered to the tenets of the Declaration of Helsinki. Ethical approval was obtained for all studies. The studies which constituted Covid datasets, Head and Neck dataset, Sarcopenia dataset and Uterine masses dataset were approved by Institutional Review Board Comité d’éthique de la recherche APHP.5 (previously CERAPHP.5, CERAPHP.Centre IRB00011928), which waived the need for written informed consent. The study which constituted Orbital Lesion dataset was approved by Comité d'Éthique pour la Recherche Hôpital Fondation Rothschild (IRB00012801) and signed informed consent was obtained from all subjects.

These datasets included radiomics features extracted from different imaging modalities addressing various diagnostic questions. All diagnoses were binary. Datasets included between 97 and 693 patients and between 105 and 606 radiomics features per sample (Table 1). One dataset included five different segmented Regions Of Interest (ROI) and another two different ROIs extracted from the same sets of images. The others included a single ROI per image.

**Table 1:** Description of the datasets used.

Diagnostic questions	Region of Interest	Number of images	Number of patients	Number of features	Prevalence (%)	Imaging modality	Multicentric
Covid severity	Heart	693	693	107	20	CT	Y
	Right Lung (total)						
	Left Lung (total)						
	Right lung lesion						
	Left lung lesion						
Sarcopenia	Psoas muscle	180	111	159	42	CT	Y
	Posterior muscle	179	110	159			
Benign vs malignant	Orbital lesions	200	175	606	37	MRI	N
Benign vs malignant	Uterine masses	167	167	315	26	MRI	Y
HPV status	Head and neck cancers	96	96	105	36	MRI	Y

The COVID severity dataset was a set of CT images from a multicentric database^3^ in which ROIs were defined in lungs to quantify severity of infection, and in the mediastinum to determine whether cardiac comorbidities affected prognosis. The sarcopenia dataset was a set of CT images from a multicentric database^5^ in which ROIs were defined on psoas and posterior muscles at L3 level to quantify muscle surface. Orbital lesions^4^, Uterine masses^6^, and Head and Neck cancers (unpublished data) were MRI datasets in which ROIs were drawn on tumors respectively for tumor characterization (benign vs malignant) or to correlate to tumor biology.

*CT* computed tomography, *MRI* magnetic resonance imaging, *HPV* human papillomavirus, *Y* yes, *N* No.

## Methods

### Evaluation of performances of algorithms

We selected the following seven algorithms most often used in radiomics studies for feature selection, based on filtering approaches. These filters can be grouped into three categories : those from the statistical field including the Pearson correlation coefficient (abbreviated as “Pearson” in the manuscript) and Spearman correlation coefficient (“Spearman “), those based on random forests including Random Forest Variable Importance (“RfVarImp “) and Random Forest Permutation Importance (“RfPerImp”), and those based on the information theory including Joint Mutual Information (“JMI”), Joint Mutual Information Maximization (“JMIM”) and Minimum-Redundancy-Maximum-Relevance (“MRMR”).

These methods rank features, and then a given number of best features are kept for modeling. Three different numbers of selected features were investigated in this study: 10, 20 and 30.

Moreover, in order to estimate the impact of the feature selection step, two non-informative algorithms of feature selection were used as benchmarks: no selection which resulted in selecting all features (“All”) and a random selection of a given number of features (“Random”).

Fourteen machine-learning or statistical binary classifiers were tested, among those most often used in radiomics studies: K-Nearest Neighbors (“KNN”); five linear models including Linear Regression (“Lr”), three Penalized Linear Regression (Lasso Penalized Linear Regression (‘LrL1”), Ridge Penalized Linear Regression (“LrL2″), Elastic-net Linear Regression (“LrElasticNet”)) and Linear Discriminant Analysis (“LDA”); Random Forest (“RF”); AdaBoost and XGBoost; three support vector classifiers including Linear Support Vector Classifier (“Linear SVC”), Polynomial Support Vector Classifier (“PolySVC”) and Radial Support Vector Classifier (“RSVC”); and two bayesian classifiers including Binomial Naive Bayes (“BNB”) and Gaussian Naive Bayes (“GNB”).

In order to estimate performances of each of the 126 combinations of the nine feature selection algorithms with the fourteen classification algorithms, each combination was trained using a grid-search and nested cross validation strategy^15^ as follows.

First, datasets were randomly split into three folds, stratified on the diagnostic value so that each fold had the same diagnostic distribution as the population of interest. Each fold was used in turn as the test set while the two remaining folds were used as training and cross-validation sets.

Ten-fold cross validation and grid-search were used on the training set to tune the hyperparameters maximizing the area under the receiver operating characteristic curve (AUC). Best hyperparameters were then used to train the model on the whole training set.

In order to take into account overfitting, the metric used was the AUC penalized by the absolute value of the difference between the AUCs of the test set and the train set:

$${\text{AUC}}_{{{\text{Cross}} - {\text{Validation}}}} = {\text{AUC}}_{{{\text{Test}} - {\text{Fold}}}} - \left| {{\text{AUC}}_{{{\text{Test}} - {\text{Fold}}}} - {\text{AUC}}_{{{\text{Train}} - {\text{Fold}}}} } \right|$$

This procedure was repeated for each of the ten datasets, for three different train-test splits and the three different numbers of selected features.

Each combination of algorithms yielded 90 (3 × 3 × 10) AUCs, apart from combinations using the “All” feature selection which were associated with only 30 AUCs due to the absence of number of feature selection, the “Random” feature selection, repeated three times which yielded 270 AUCs. Hence, in total, 13,020 AUCs were calculated.

### Statistical analysis

Multifactor ANalysis of VAriance (ANOVA) was used to quantify the variability of the AUC associated with the following factors: dataset, feature selection algorithm, classifier algorithm, number of features, train-test split, imaging modality, and interactions between classifier / dataset, classifier / feature selection, dataset / feature selection, and classifier / feature selection / dataset. Proportion of variance explained was used to quantify impacts of each factor/interaction. Results are given as frequency (proportion(%)) or range (minimum value; maximum value).

For each feature selection, classifier, dataset and train-test split, median AUC,1^st^ quartile (Q1); and 3^rd^ quartile (Q3) were computed. Box-plots were used to visualize results.

In addition, for feature selection algorithms and classifiers, a Friedman test^16^ followed by post-hoc pair-wise Nemenyi-Friedman tests were used to compare the median AUCs of the algorithms.

Heatmaps were generated to illustrate results for each Feature Selection and Classifier combination.

### Implementation

All the algorithms were implemented in Python (version 3.8.8). Pearson and Spearman correlations were computed using Pandas (1.2.4), the XGBoost algorithm using xgboost (1.5) and JMI, JMIM and MRMR algorithms using MIFS. All other algorithms were implemented using the scikit-learn library (version 0.24.1). Data were standardized by centering and scaling using scikit-learn StandardScaler.

## Results

AUCs ranged from 0.20 to 0.91 when considering all possible combinations. Four hundred thirty-five (3.4%) AUCs were below 0.5.

**Figure 1:**
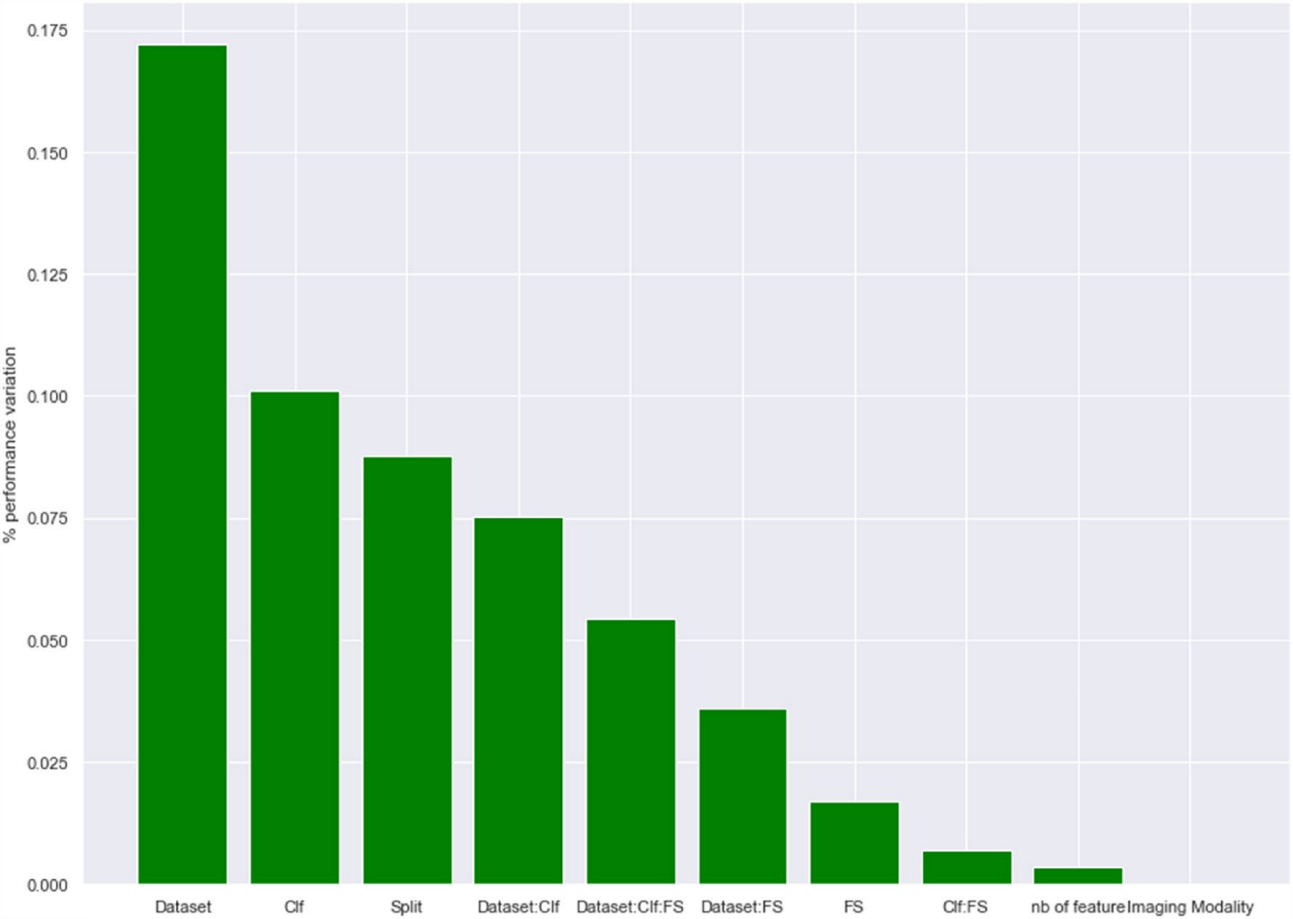
Proportion of performance variation explained by dataset and model property. There remained 45% of variation which was not explained by factors represented. Clf : classifier, FS : feature selection, “:” represents interaction between factors.

Figure 1 shows proportion of performance variation explained by experimental factors. Running the multifactor ANOVA on the AUCs, the identified factors and their interactions explained 55% of the variation in modeling performance. Among these 55%, the most important factor was the dataset itself (17% of the variations), then the classifier (10%), and the train-test split (9%). The feature selection algorithm only explained 2% of the variations. Both number of selected features and imaging modality (CT vs MRI) explained less than 1% of the variation in performances. Interactions between factors explained the remaining 17%.

Table 2 shows the median [Q1;Q3] AUC for each of the feature selection algorithms, regardless of the classifier used. Differences in median AUCs were slight between all possible combinations, ranging from 0.68 to 0.70, yet were statistically significantly different (*P*-value < 1e−32). Pairwise comparisons are presented in SI Table 1.

**Table 2:** AUC performances for Feature Selection algorithms displayed from lowest to highest median value.

Feature selection	Median	Q1	Q3
Random	0.675	0.615	0.719
RFVarImp	0.677	0.624	0.722
Spearman	0.678	0.613	0.724
Pearson	0.682	0.620	0.725
RFPermImp	0.683	0.611	0.731
All	0.695	0.636	0.731
MRMR	0.696	0.643	0.742
JMIM	0.701	0.654	0.746
JMI	0.703	0.650	0.748

Random : Random Selection of features (non-informative) ; Pearson : Pearson correlation coefficient; Spearman: Spearman correlation coefficient; RfVarImp : Random Forest Variable Importance; RfPermImp : Random Forest Permutation Importance; JMI : Joint Mutual Information; JMIM : Joint Mutual Information Maximization; MRMR : Minimum-Redundancy-Maximum-Relevance.

Information theory algorithms (JMI and JMIM) had the highest values. All : No-Selection of features (non-informative).

Feature selection algorithms based on information theory such as JMI and JMIM provided the best overall performances as seen with their higher median AUC at 0.70 respectively and their relatively high Q1, ensuring consistently good performances. All feature selection algorithms performed better than the “Random” feature selection.

Table 3 shows the median [Q1;Q3] AUC for each of the classifier algorithms, regardless of the feature selection used. The difference between median AUC of classifier algorithms was significant (*P*-value < 1e−32). Pairwise comparisons are presented in SI Table 2.

**Table 3:** AUC performances for classifier algorithms displayed from lowest to highest median value.

Classifier	Median	Q1	Q3
polySVC	0.619	0.532	0.690
RSVC	0.659	0.588	0.706
linearSVC	0.663	0.580	0.724
KNN	0.663	0.612	0.712
AdaBoost	0.671	0.622	0.718
XGBoost	0.680	0.628	0.719
BNB	0.688	0.640	0.724
lr	0.690	0.641	0.729
lrl1	0.694	0.604	0.748
GNB	0.698	0.648	0.733
lrElasticNet	0.706	0.654	0.753
rf	0.706	0.662	0.740
lda	0.707	0.660	0.748
lrl2	0.710	0.661	0.749

KNN:K-Nearest Neighbors; Lr : Linear Regression; LrL1 : Lasso Penalized Linear Regression; LrL2 : Ridge Penalized Linear Regression; LrElasticNet : Elastic-net Linear Regression; LDA : Linear Discriminant Analysis; RF : Random Forest; AdaBoost : AdaBoost; XGBoost : XGBoost; Linear SVC : Linear Support Vector Classifier; Poly SVC : Polynomial Support Vector Classifier; RBFSVC : Radial Support Vector Classifier; BNB : Binomial Naive Bayes; GNB : Gaussian Naive Bayes.

On our datasets, Linear classifier algorithms (Ridge Penalized Linear Regression, Elastic-net Linear Regression, Linear Discriminant Analysis) and Random Forest gave consistently better performances (median AUCs greater than 0.70). Some algorithms, such as KNN, AdaBoost or XGBoost, gave lower overall performances, though they could reach occasionally very high performances on some combinations of dataset/number of features/train-test split.

**Figure 2:**
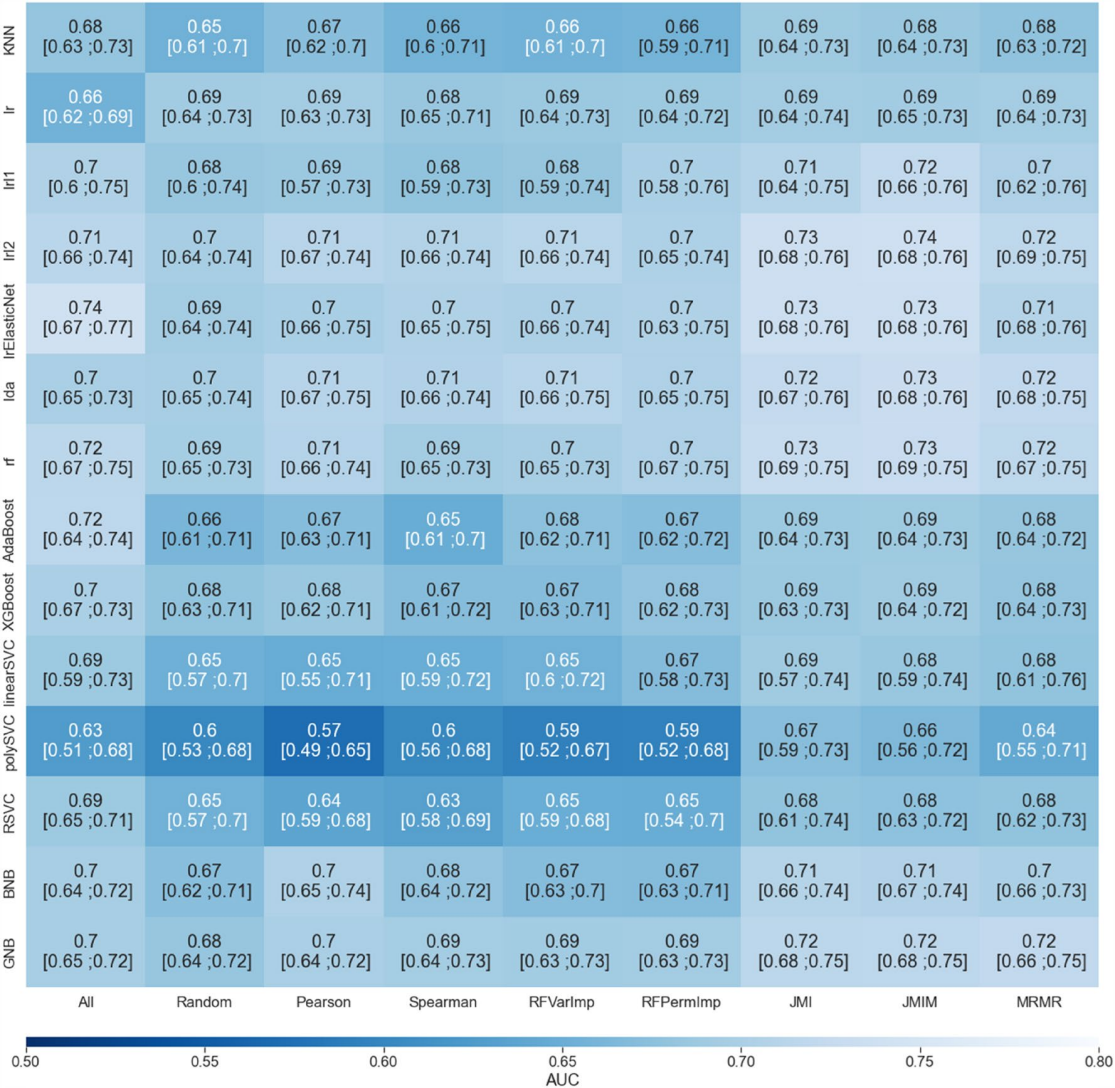
Heat map of median [Q1; Q3] AUC scores for all 9 × 14 combinations of feature selection algorithms and classifiers. All : No feature selection (non-informative); Random : Random feature selection (non-informative) ; Pearson : Pearson correlation coefficient; Spearman : Spearman correlation coefficient; RfVarImp : Random Forest Variable Importance; RfPermImp : Random Forest Permutation Importance; JMI : Joint Mutual Information; JMIM : Joint Mutual Information Maximization; MRMR : Minimum-Redundancy-Maximum-Relevance ; KNN:K-Nearest Neighbors; Lr : Linear Regression; LrL1 : Lasso Penalized Linear Regression; LrL2 : Ridge Penalized Linear Regression; LrElasticNet : Elastic-net Linear Regression; LDA : Linear Discriminant Analysis; RF: Random Forest; AdaBoost : AdaBoost; XGBoost : XGBoost; Linear SVC : Linear Support Vector Classifier; Poly SVC : Polynomial Support Vector Classifier; RBFSVC : Radial Support Vector Classifier; BNB : Binomial Naive Bayes; GNB : Gaussian Naive Bayes.

Figure 2 shows the heatmap of median AUC for all feature selection algorithms and classifiers. Median AUC ranged between 0.57 and 0.74. With the exception of the combination None-lrElasticNet, the best combinations of algorithms were those using best feature selection algorithms (JMI, JMIM, MRMR) and best classifier algorithms (penalized linear regressions and Random Forest).

**Figure 3:**
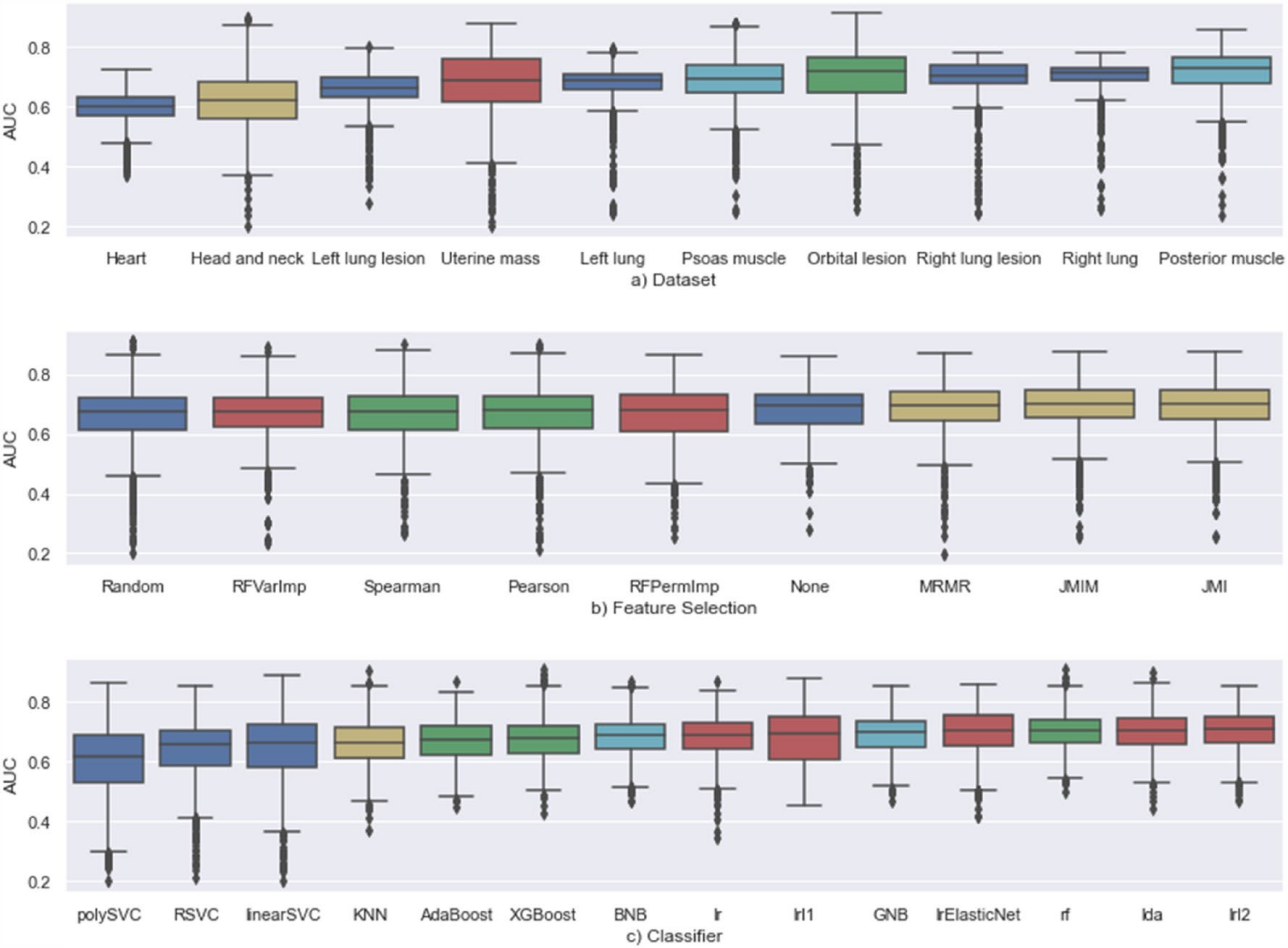
Boxplot of AUCs by (**a**) dataset, (**b**) feature selection algorithm and (**c**) classifier. All : No-Selection of features (non-informative); Random : Random Selection of features (non-informative) ; Pearson : Pearson correlation coefficient; Spearman : Spearman correlation coefficient; RfVarImp : Random Forest Variable Importance; RfPermImp : Random Forest Permutation Importance; JMI : Joint Mutual Information; JMIM : Joint Mutual Information Maximization; MRMR : Minimum-Redundancy-Maximum-Relevance ; KNN:K-Nearest Neighbors; Lr : Linear Regression; LrL1 : Lasso Penalized Linear Regression; LrL2 : Ridge Penalized Linear Regression; LrElasticNet : Elastic-net Linear Regression; LDA : Linear Discriminant Analysis; RF : Random Forest; AdaBoost : AdaBoost; XGBoost : XGBoost; Linear SVC : Linear Support Vector Classifier; Poly SVC : Polynomial Support Vector Classifier; RBFSVC : Radial Support Vector Classifier; BNB : Binomial Naive Bayes; GNB : Gaussian Naive Bayes.

Figure 3 shows box-plots of AUCs for the different datasets, feature selection and classifier algorithms. The Covid severity dataset provided smaller distributions of AUCs.

**Figure 4:**
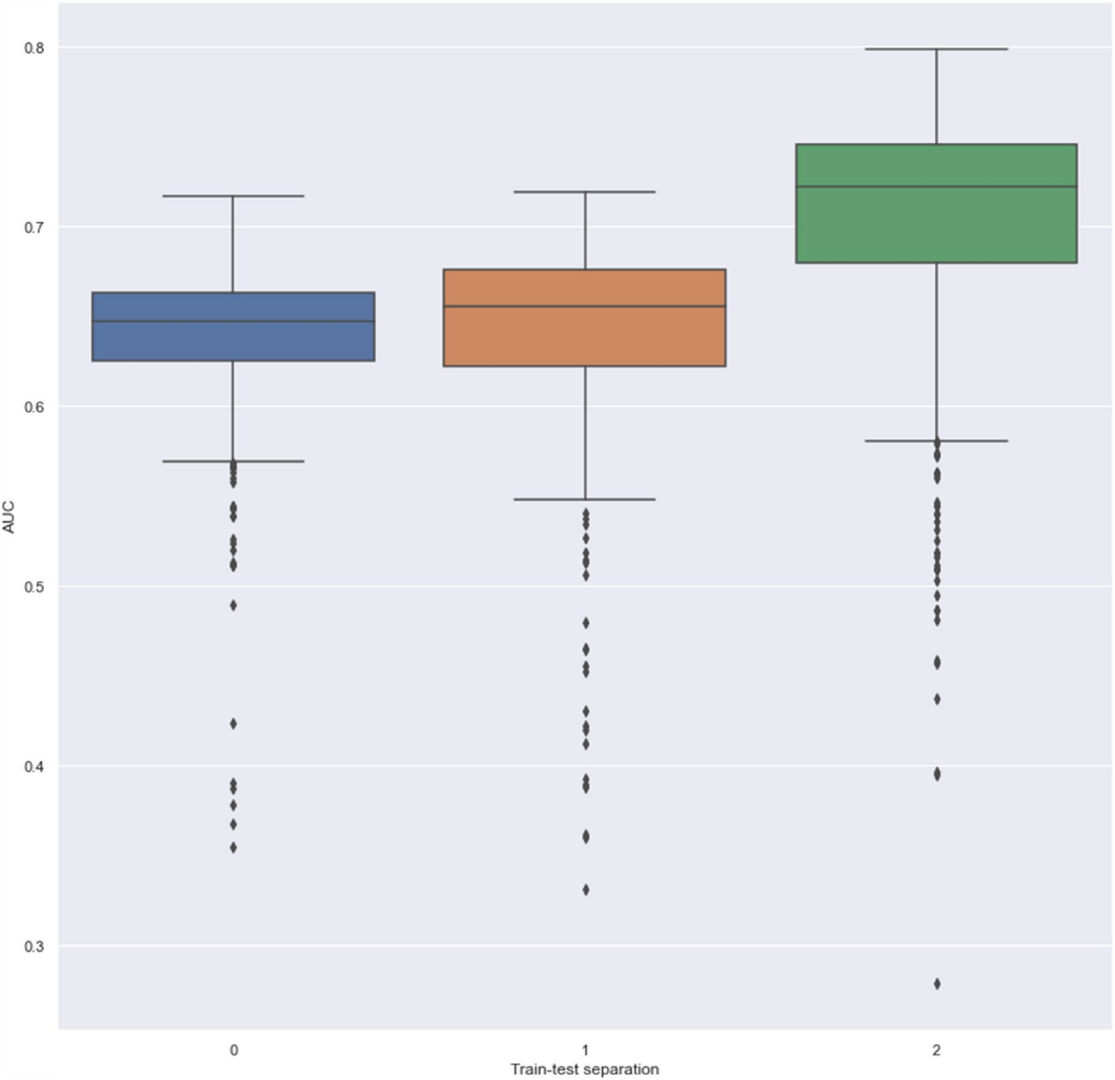
Boxplot of AUCs for the different train-test splits of the “Left lung” dataset. Respective percentage of the high severity class of COVID disease in the three test datasets were 82, 78 and 80%.

Figure 4 shows the boxplots of AUC for the different train-test split separation of left lung lesion dataset, as an example. Boxplots for the other datasets are given in SI Fig. 1–9. Maximum difference in median AUC between the train and the test performance was 0.11 on the Head and neck dataset while minimum difference was 0.00 on the right lung ROI from the COVID dataset.

## Discussion

In this study, we compared combinations of feature selection algorithms and classifiers in ten different datasets. Firstly, the factor most impacting variations in performance was the dataset itself, probably reflecting the quantity of information truly present in the data. Secondly, feature selection algorithms based on information theory performed consistently higher than other algorithms, for any given dataset. However, the choice of the feature selection algorithm had little effect on performance when analyzing variations using ANOVA. Thirdly, for a given dataset, choice of classifiers was the most impacting factor. Some classifiers performed generally better (Random Forest, Linear Discriminant Analysis and Ridge Penalized Linear Regression), however there was no algorithm that consistently gave the best performance. Finally, the train-test split explained 9% of the variations in performance.

Our study finds similar results to previous publications. Two main studies investigated the impact of algorithm choice on performances in radiomics, Parmar et al. on 464 lung cancer CT^8^ and Sun et al. on 285 brain MRI in glioblastoma^17^. In Parmar’s study, the classifier was the most important source of variability of performance, similar to our study. Random Forest gave the best result in Parmar’s study, while LDA gave the best result in Sun’s study, both of which are also consistent with our results. Studies in other research fields also supply insight for radiomics. Wang and Liu’s study on microbiology used 29 datasets which include between 29 and 512 observations^18^. In this study SVC provided poorer results than Elastic-net, Random Forest or XGBoost. These results could be explained by the similarity between radiomics and microbiology datasets in terms of number of observations and number of available features.

Feature selection seemed to have a smaller impact on performances in our study compared to that of Parmar, but results of the ANOVA showed that there was an interaction between feature selection algorithms and dataset implying that some feature selection algorithms appeared more adapted to some datasets. This may explain why the best feature selection algorithms varied in the different studies because they were applied to single datasets^17,19^. Information theory-based algorithms may perform better because they take into account the potential redundancy between features as well as the information brought by the feature. Regarding the number of features selected, Parmar^19^ and Sun^17^ are in line with our results showing the low impact on performance.

This study highlights some factors explaining variability in performances in radiomics. Datasets usually contain a number of features far greater than independent observations, and even with dimension reduction, this leads to overfitted models and poor generalizability. Radiomics models are often evaluated using a train-test strategy. However, radiomics studies, including our own, show that different train-test splits may lead to variations in performances. An et al. studied the impact of the train-test strategy on 258 meningioma MRIs and showed that using a single random train-test split led to a loss in performance (generalization gap) when applied to a test dataset, especially with small datasets and when working on a difficult task^20^. Studies on Gaussian data showed that nested cross-validation is a better way to evaluate model performances. Varma and Simon showed cross-validation underestimated the true error of a model by more than 20% in one out of five simulations^21^. Vabalas et al. also investigated five validation approaches on simulated Gaussian data. They showed cross-validation could lead to over-fitting by reusing the data in both training and validation folds, whereas nested cross-validation led to a smaller bias. The impact of the train-test split is probably due to the relatively low number of samples in each dataset compared to biological variability. It results in performances being highly susceptible to the distribution of data in the training vs the test set and may partly explain lack of generalizability of results that may be observed in published radiomics studies. To compensate for the impact of the train-test split, a nested cross validation could be used. This strategy is rarely used in radiomics studies, and we believe it could improve performances of discovered signatures when applied to an external validation dataset.

When performing radiomics studies in a specific dataset, a common strategy is to simultaneously test several combinations of feature selection algorithms and classifiers to choose the one that optimizes performance. Indeed, a large number of feature selection algorithms and classifiers are available. However, multiplying the number of models tested may lead to an increase in the rate of overfitting and false discoveries, similar to false discovery rates observed in genomics. Based on our results, it might be more efficient to select a smaller number of combinations, for a better balance between optimization and overfitting. This would also reduce computation time. Similar to other scientific benchmarks, algorithms with the same underlying approaches seem to give similar results^22^. When determining which smaller subset of models should be tested in a radiomics study, one strategy therefore could be to choose classifiers from different families. The overall number of algorithms that should be tested in a single dataset is not defined, however, and may also depend on available computation time and dataset size. Determining the right number of algorithms was out of the scope of this study but should be further investigated. 

There are some limits to our study. While most radiomics studies focus on a single dataset, our work analyzed ten datasets from previously published radiomics studies, which strengthened the generalizability of our results. However, dataset characteristics were similar, in particular regarding the number of observations and prevalence. Thus, the impact of dataset characteristics could not be fully investigated in this study. Though it was not possible to compute the exact portion of variation explained by dataset characteristics, we hypothesize that it contributed in part to the explained 17% in modeling performance variation and possibly to some of the remaining unexplained 45% variation. Though we investigated the impact of the train-test split on performances, few iterations were done to estimate the impact of randomness during the train-test split, which prevented us from estimating precisely the impact of chance at this step. Finally, as in every analysis of variance, a portion of the unexplained variation in modeling performance might be related to unobserved, possibly unobservable, characteristics. Identification of some of the unobserved parameters in our study would be a useful step toward increasing the explained portion of variation in modeling performance.

Another limitation of the present study was the relatively small number of algorithms tested. Only seven feature selection algorithms and fourteen classifiers were investigated, which is only a small portion of the large number of available algorithms. Though linear methods provided good performances, non-linear feature transformation^23^ or wrapper feature selection algorithms may have improved performances. However, its implementation was beyond the scope of this study which was meant to focus on filter feature selection, most often used in radiomics studies. Finally, neural networks were not used, in part due to the small datasets.

## Conclusion

When performing radiomics, model performances may vary greatly and these variations are related to several main factors, including the dataset itself, the type of classifier and the split between train and test subsets. We recommend testing a small number of feature selection and classifier combinations to avoid false discovery due to multiple testing and overfitting. Feature selection algorithms based on information theory on the one hand, and penalized linear models and random forest as classifiers on the other hand seemed to perform the most consistently across datasets.

### Supplementary Information

Supplementary Information.

## Data Availability

Datasets are not publicly available. Data access is subject to each dataset’s specific ethical authorizations for secondary use and may be submitted to the corresponding author.
