# Supplementary material for: Comparative performances of machine learning algorithms in radiomics and impacting factors
Source: Sci Rep. 2023 Aug 28;13:14069. doi: 10.1038/s41598-023-39738-7 (PMC10462640; doi:10.1038/s41598-023-39738-7)
Supplement: Supplementary file 1 — Supplementary Information. [file 41598_2023_39738_MOESM1_ESM.docx]

##
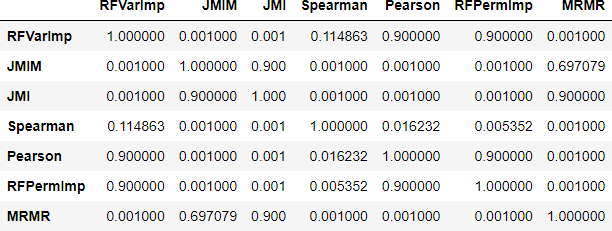
Supplementary material

## SI Table 1 : Table of p-value of pair-wise Nemenyi test for Feature Selection Algorithms. Pearson : Pearson correlation coefficient; Spearman : Spearman correlation coefficient; RfVarImp : Random Forest Variable Importance; RfPermImp : Random Forest Permutation Importance; JMI : Joint Mutual Information; JMIM : Joint Mutual Information Maximization; MRMR : Minimum-Redundancy-Maximum-Relevance.

##
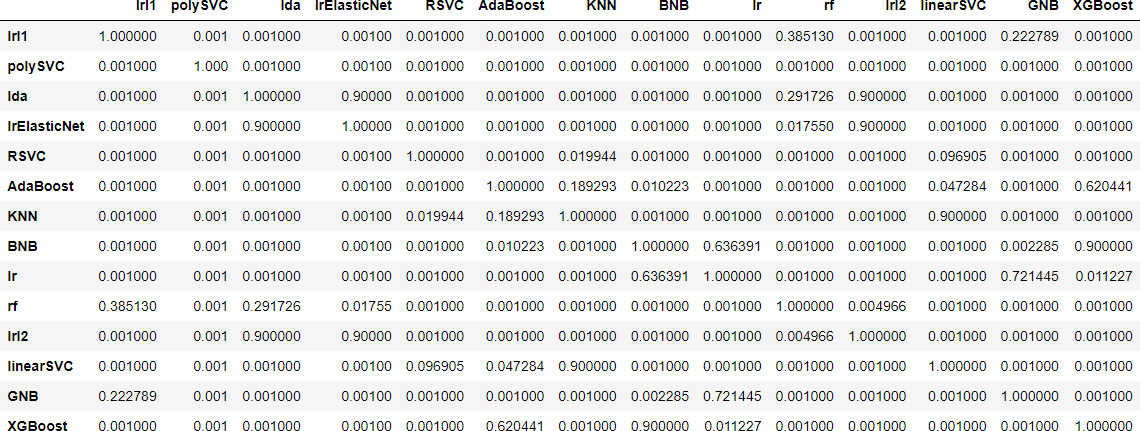
SI Table 2 : Table of p-value of pair-wise Nemenyi test for Classifier Algorithms. KNN:K-Nearest Neighbors; Lr : Linear Regression; LrL1 : Lasso Penalized Linear Regression; LrL2 : Ridge Penalized Linear Regression; LrElasticNet : Elastic-net Linear Regression; LDA : Linear Discriminant Analysis; RF : Random Forest; AdaBoost : AdaBoost; XGBoost : XGBoost; Linear SVC : Linear Support Vector Classifier; Poly SVC : Polynomial Support Vector Classifier; RBFSVC : Radial Support Vector Classifier; BNB : Binomial Naive Bayes; GNB : Gaussian Naive Bayes.

##
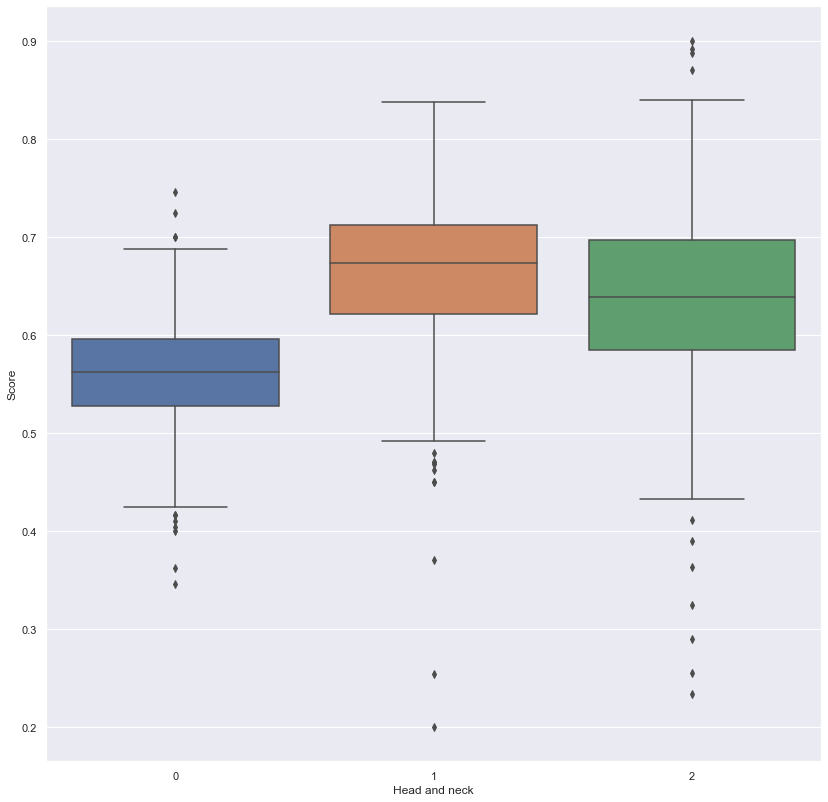

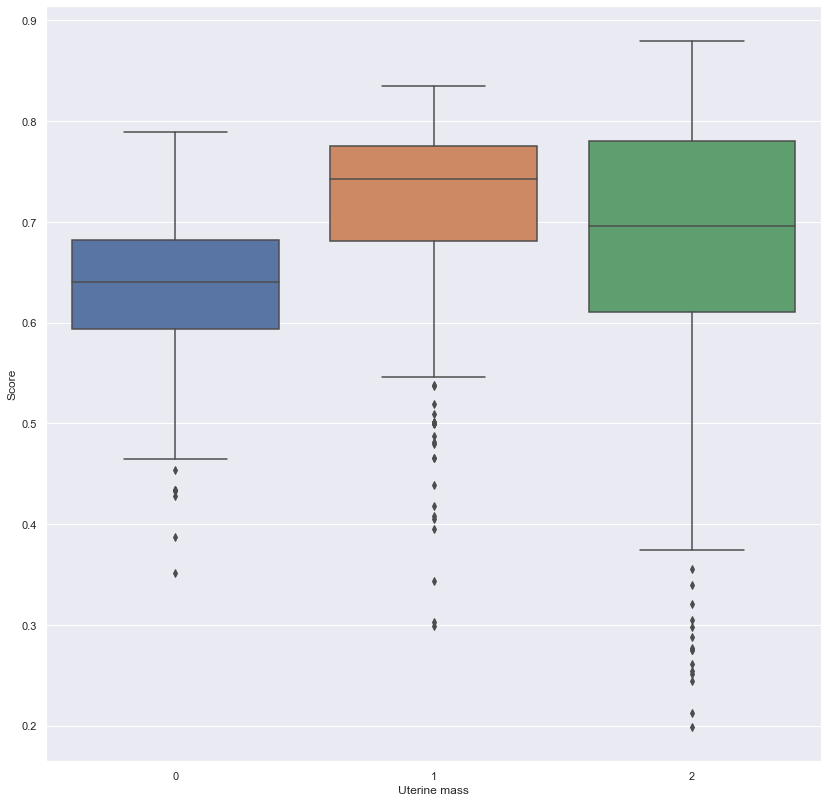

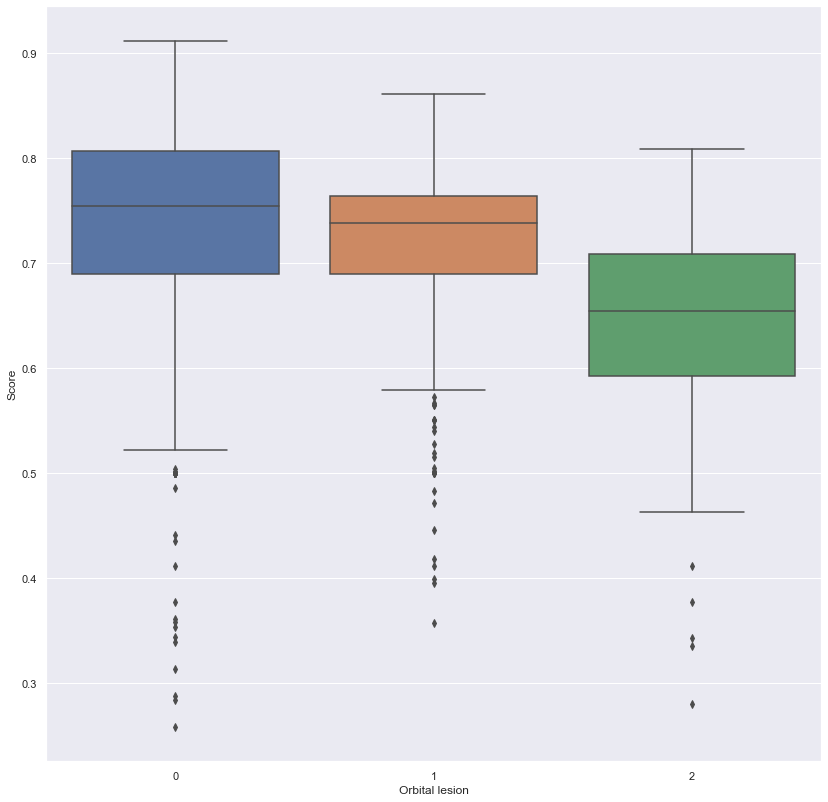

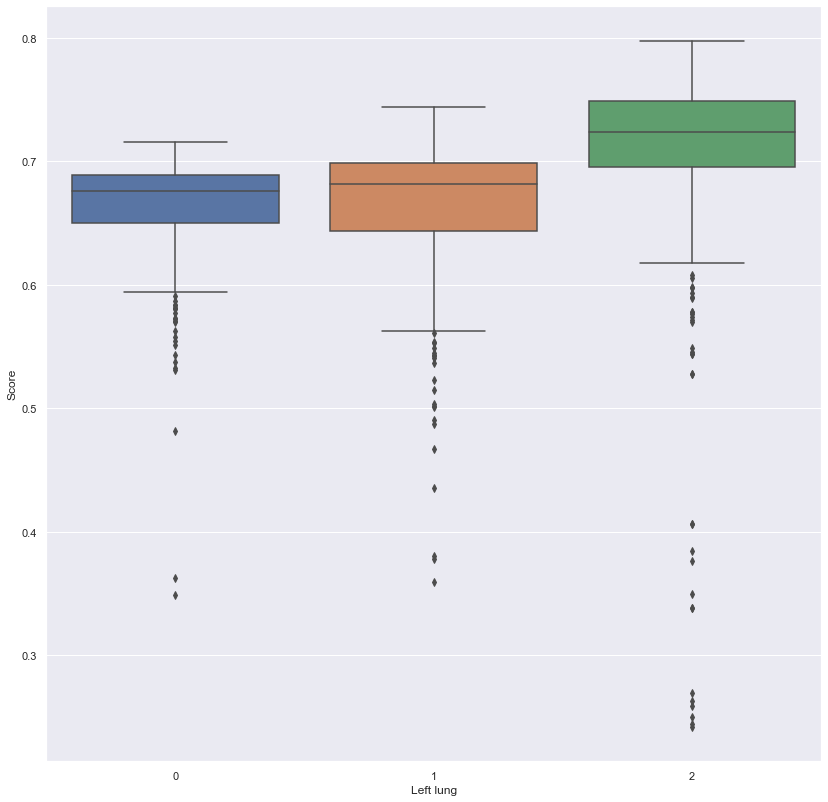
SI Figure 1 :Boxplot of AUCs for the different train-test split separation of uterine mass dataset.

**
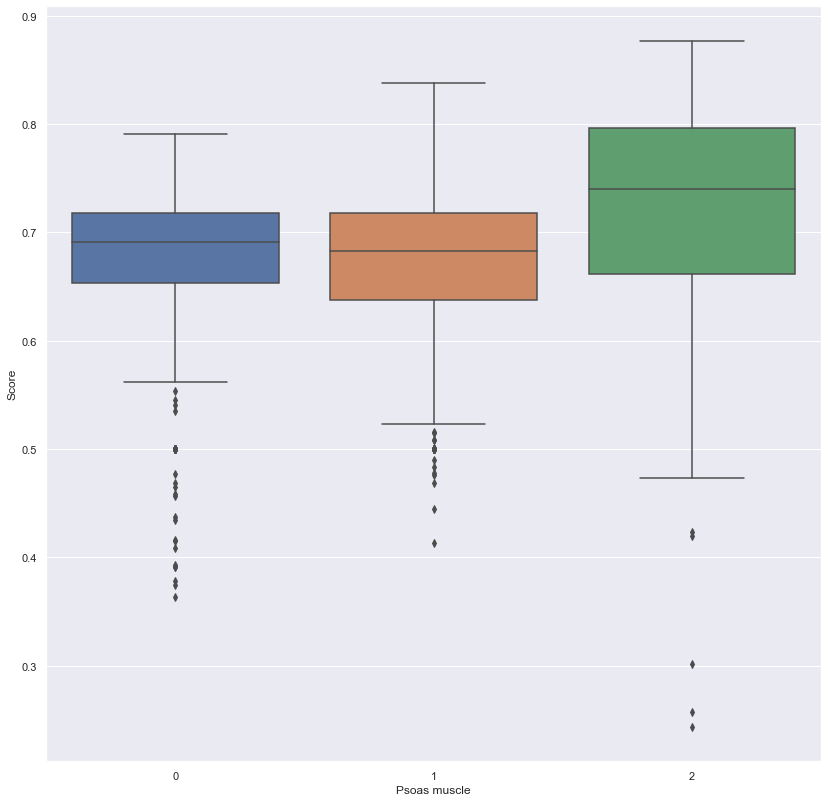
SI Figure 2 :Boxplot of AUCs for the different train-test split separation of psoas muscle dataset.**

**
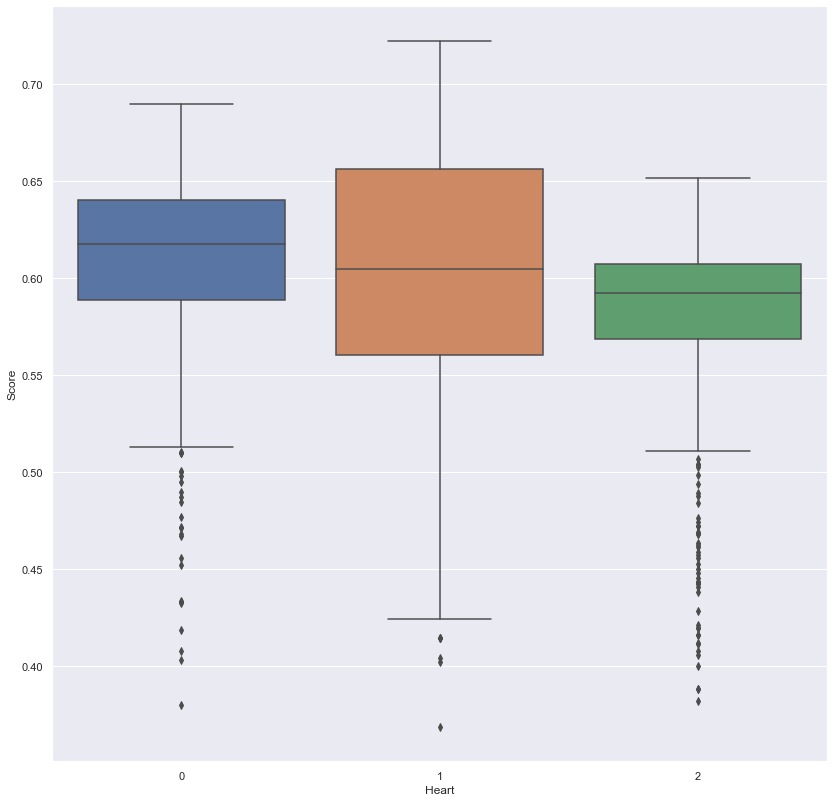
SI Figure 3 :Boxplot of AUCs for the different train-test split separation of Heart dataset.**

**
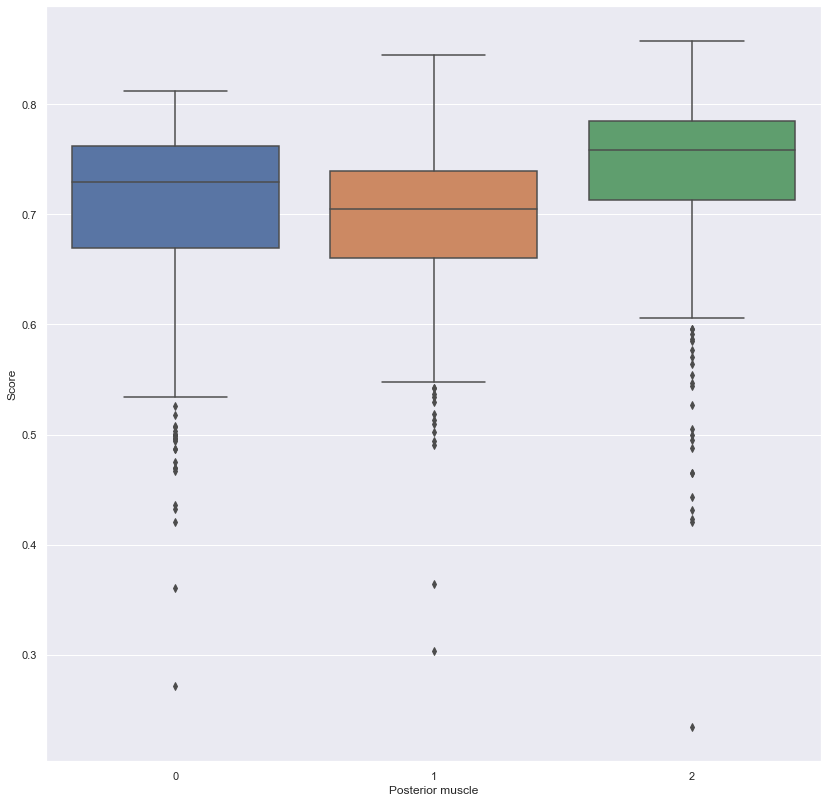
SI Figure 4 :Boxplot of AUCs for the different train-test split separation of posterior dataset.**

**
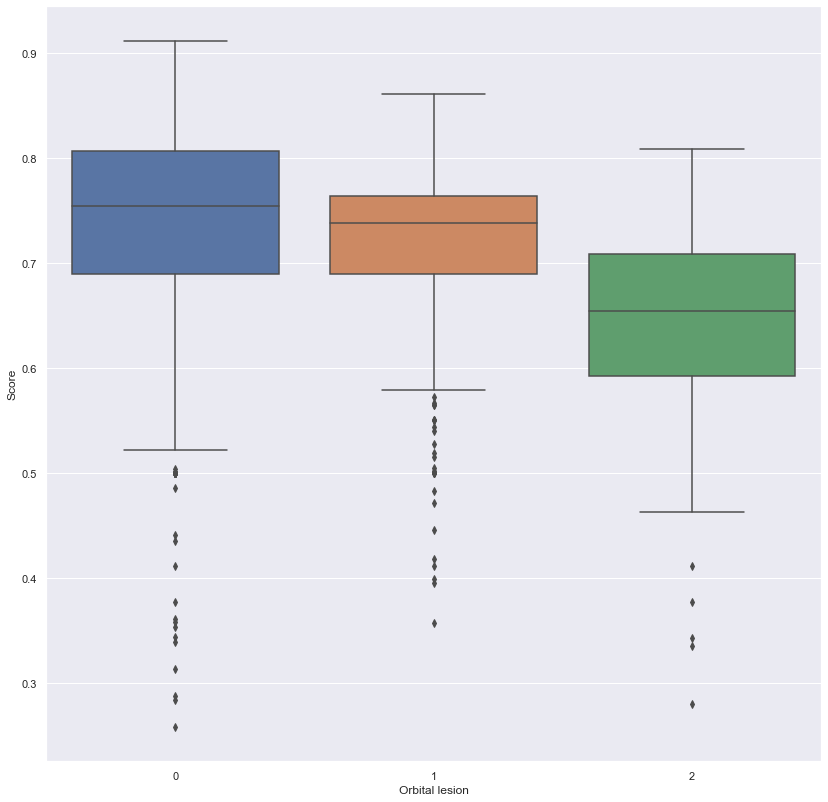
SI Figure 5 :Boxplot of AUCs for the different train-test split separation of orbital lesion dataset.**

**
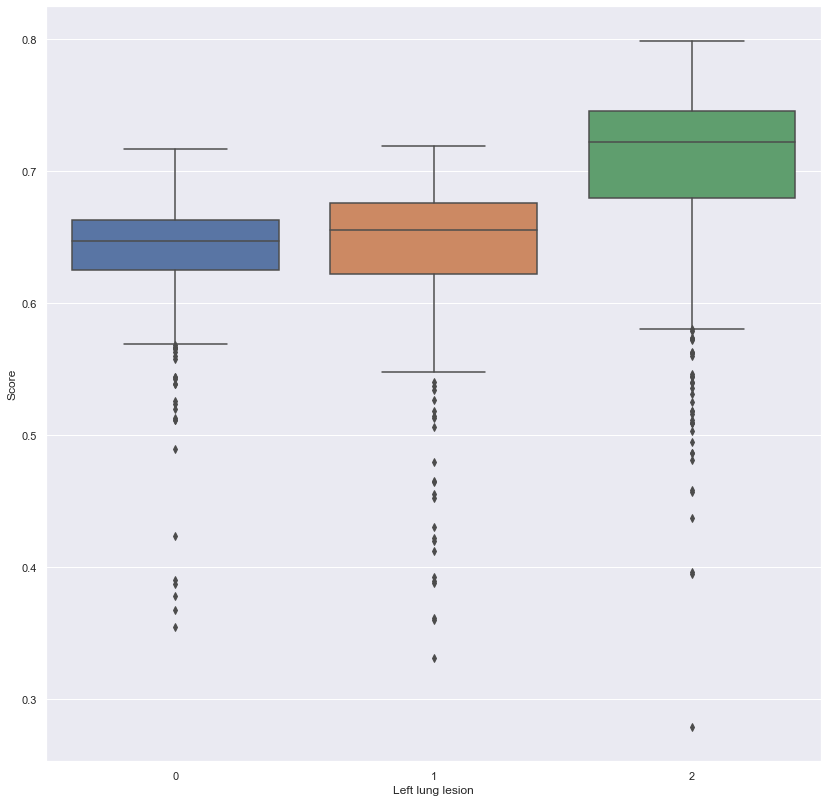
SI Figure 6 :Boxplot of AUCs for the different train-test split separation of left lung lesion dataset.**

**
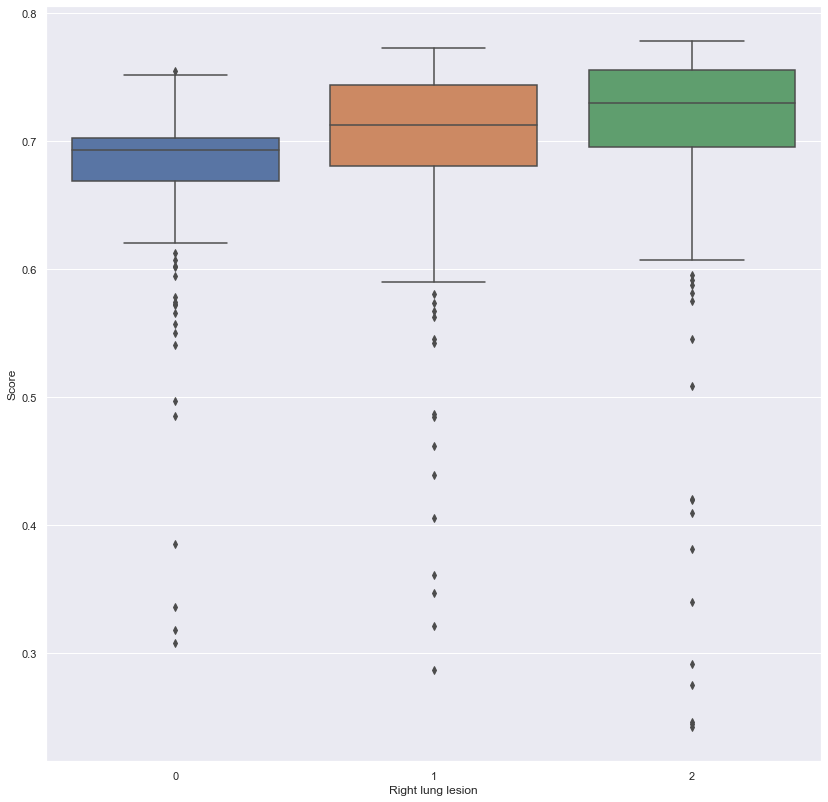
SI Figure 7 :Boxplot of AUCs for the different train-test split separation of right lung lesion dataset.**

**
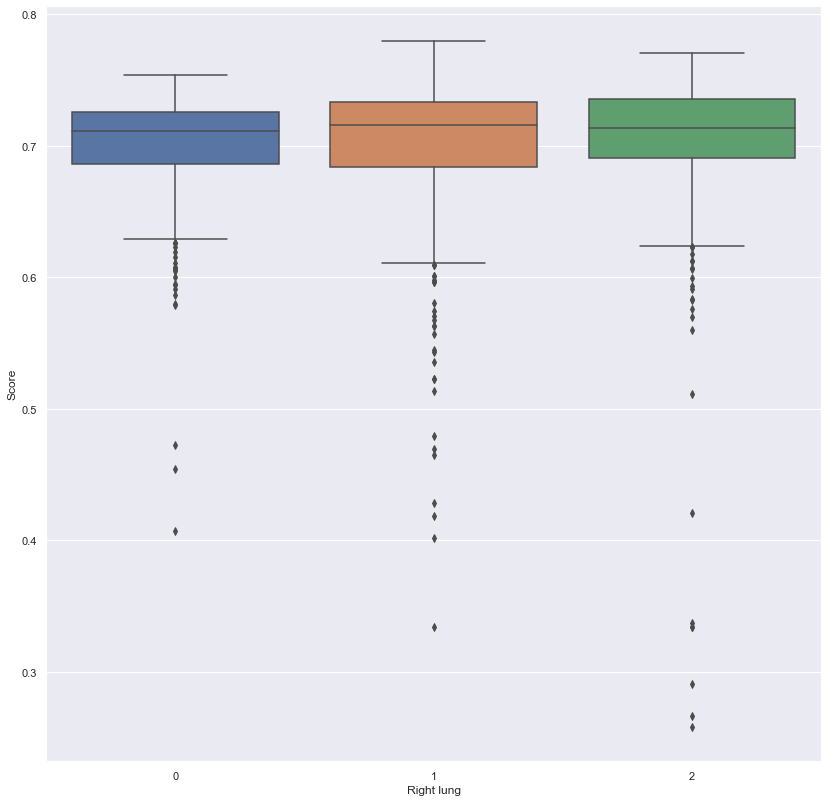
SI Figure 8 :Boxplot of AUCs for the different train-test split separation of right lung dataset.**

**
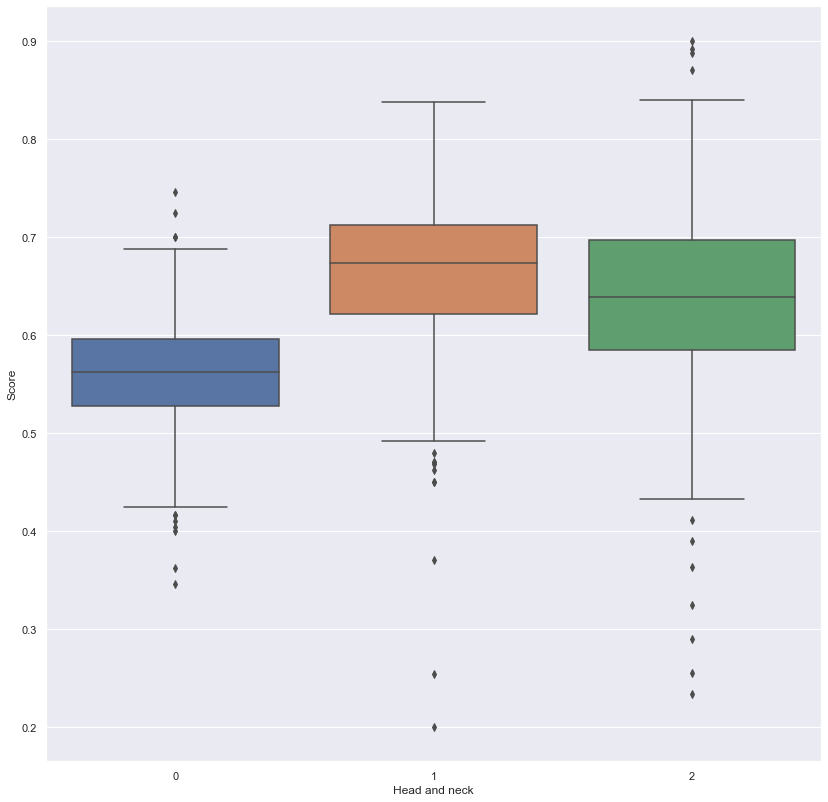
SI Figure 9 :Boxplot of AUCs for the different train-test split separation of head and neck dataset.**
